# Glastonbury Festival: Medical Care at the World’s Largest Greenfield Music Festival

**DOI:** 10.1017/S1049023X24000256

**Published:** 2024-04

**Authors:** Jack F. Bennett, David J. Cottrell

**Affiliations:** 1. School of Medicine, University of Leeds, Leeds, United Kingdom; 2. Department of Diabetes and Endocrinology, Liverpool University Hospitals NHS Foundation Trust, Liverpool, United Kingdom

**Keywords:** Emergency Medical Services, event medicine, mass-gathering event, music festival

## Abstract

**Introduction:**

Music festivals have become an increasingly popular form of mass-gathering event, drawing an increasing number of attendees across the world each year. While festivals exist to provide guests with an enjoyable experience, there have been instances of serious illness, injury, and in some cases death. Large crowds, prolonged exposure to loud music, and high rates of drug and alcohol consumption can pose a dangerous environment for guests as well as those looking after them.

**Methods:**

A retrospective review of electronic patient records (EPRs) at the 2022 Glastonbury Festival was undertaken. All patients who attended medical services on-site during the festival and immediately after were included. Patient demographics, diagnosis, treatment received, and discharge destination were obtained and analyzed.

**Results:**

A total of 2,828 patients received on-site medical care. The patient presentation rate (PPR) was 13.47 and the transport-to-hospital rate (TTHR) was 0.30 per 1,000 guests. The most common diagnoses were joint injuries, gastrointestinal conditions, and blisters. Only 164 patients (5.48%) were diagnosed as being intoxicated. Overall, 552 patients (19.52%) were prescribed a medication to take away and 268 (9.48%) had a dressing for a minor wound. One patient (0.04%) underwent a general anesthetic and no patients required cardiopulmonary resuscitation. Most patients were discharged back to the festival site (2,563; 90.66%).

**Discussion:**

Minor conditions were responsible for many presentations and most patients only required mild or non-invasive interventions, after which they could be safely discharged back to the festival. Older adults were diagnosed with a different frequency of conditions compared to the overall study population, something not reported previously. Intoxicated patients only accounted for a very small amount of the medical workload.

## Introduction

Music festivals are a type of mass-gathering event that are attended by millions around the world each year. Offering live music, cultural displays, and performing arts, they have seen a substantial rise in popularity and are now thought to be attended by over one-quarter of adults within the United Kingdom.^1^ Whilst aiming to provide their guests with an enjoyable experience, they have also been associated with negative health outcomes.^2^

The literature has reported patients presenting with a wide variety of injuries and illnesses that have occurred at previous music festivals from minor illness to trauma, intoxication, and even death.^3–5^ In a review of grey literature, Turris and Lund reported 722 deaths at music festivals from 1999 through 2014.^6^ They found 82% of all deaths were from traumatic causes whilst only 13% were from a drug overdose. Despite this, the media continues to suggest the potentially deadly consequences of music festivals are only associated with illicit drugs.^7,8^

Compared to other mass-gathering events, music festivals appear to be most affected by substance use and intoxication.^9^ Previous research has shown that drug and alcohol consumption is particularly prevalent at music festivals, and this has been confirmed through urine analysis, which has revealed high levels of recreational drugs such as cocaine, ketamine, and amphetamines.^10–12^ At the 2021 Glastonbury Festival (Somerset, England), Aberg, et al showed the levels of some recreational drugs in local rivers and waterways were so high they posed a risk to the local aquatic ecosystem.^13^ Recreational drug use at music festivals can also be a significant cause of off-site discharges when patients are too unwell to be managed by medical staff at the festival, placing a strain on local health care.^14–16^

Alongside conditions attributable to the festival, research at previous music festivals has also demonstrated that medical and surgical complaints not directly related to the festival are common.^17^ Given that many festivals are in remote locations, the existing local medical infrastructure is often insufficient to support the temporary rise in population. As a result, field hospitals and medical teams are frequently deployed on-site, although they may possess varying levels of equipment and staff qualifications.^18,19^ While the deployment of qualified medical staff has been shown to reduce the rate of off-site ambulance transfers compared to first aiders alone,^5^ music festivals can still significantly impact local health care services and emergency departments may see an increase in patient attendances as a result.^14,15^

Levels of crowding, availability of drinking water, and the length of the event have also been suggested to impact the level of utilization of medical services.^2,20–22^ With festivals typically spanning several days and often taking place in remote outdoor settings, their attendees can be especially vulnerable to environmental conditions. The outdoor temperature at mass-gathering events such as music festivals has been shown to have a significant impact on the number and type of presentations.^20,23^ Given that most music festivals, including Glastonbury Festival, take place within the summer months,^24^ environmental exposure can contribute significantly to the medical burden.

The aim of this study was to present a detailed description of medical attendances at the 2022 Glastonbury Festival. Not only does Glastonbury continue to be the world’s largest greenfield music festival, but it also possesses unique characteristics compared to other festivals. Glastonbury Festival attracts a significantly wider age range of guests owing to the festival’s diverse range of performers and music genres.^24,25^ Children of any age are permitted to attend, and previous literature has already described the varied patterns of illness and injury that children may present with at mass-gathering events.^3,17^ The festival also attracts a significant number of older guests. This demographic is less commonly represented at music festivals and subsequently in the literature. Some of the genres performed at Glastonbury Festival, such as rock and hip hop, have previously been suggested to be associated with a higher number of medical complaints and traumatic injuries.^26^ Alongside these, the 2022 festival featured numerous other music genres which were simultaneously performed across a variety of stages. Concurrent performances of different genres are less common at festivals in the existing literature, so the impact of this is not yet fully known.

## Methods

This study is a retrospective observational study of electronic patient records (EPRs) at the 2022 Glastonbury Festival.

### Setting

Glastonbury Festival is set in remote countryside in Somerset, Southwest England and is spread over a 900-acre site. The festival runs annually each summer with a break every five years to allow the site to recover. The site is predominantly made up of rolling grass hills and is fully enclosed by fencing controlled by security personnel. Performing arts are on display across 31 different areas across the festival site, but most of the musical performances are on six stages concentrated in the center of the festival. There is limited shelter from the elements, with the main stage arenas of the festival completely uncovered. Some smaller stages are located in temporary tents. Alcohol is available for sale across the festival site and free water taps are readily available. Although illicit drugs are banned at the festival,^27^ previous studies have demonstrated a variety of drugs have been consumed, including cocaine and MDMA (3,4-methylenedioxymethamphetamineare).^13^ Away from the festival, there are three emergency departments located at district general hospitals 15-20 miles away.

The 2022 festival was held over five days starting Wednesday June 22, 2022 and was attended by 210,000 people.^28^ This consisted of 143,000 ticketed guests and 67,000 staff, volunteers, and performers, the majority of whom camped on the site for the duration of the festival. Adults and children of any age could attend the festival, however children under 16 must have been accompanied by an adult. The festival offered a variety of music genres, from contemporary music, rock, pop, electronic, and hip hop, to jazz, country, and folk alongside other performing arts such as comedy, theatre, and circus acts.

The were no amplified performances on Wednesday, the first day of the festival, or Monday, the last. Headline acts performed on Friday, Saturday, and Sunday, whilst smaller although amplified performances took place on Thursday.

The weather varied throughout the festival, with warm dry days at the start and cooler wet days at the end. Historic climate data from Yeovilton, approximately 12 miles away, demonstrated a range in daily maximum recorded temperature of 19.3°C (66.7°F) to 27.0°C (80.6°F) and a range in minimum temperature of 9.0°C (48.2°F) to 11.0°C (51.8°F).^29,30^ Of the five days, there was precipitation on three, ranging from 0.3mm to 3.8mm of precipitation per day.

A locally based charity provided medical cover for the festival.^31^ Alongside a primary medical center, two smaller units dispersed across the site received patients. Medical teams were also based at the two largest stages during performances. Responders on foot and on bicycles attended casualties alongside all-wheel drive ambulances. These facilities were staffed by 832 volunteers, of whom 533 were clinicians. Medical consultants in emergency medicine, anesthetics, psychiatry, radiology, and family medicine worked alongside dental surgeons, paramedics, pharmacists, podiatrists, physiotherapists, radiographers, specialist nurses, and first aiders. Ultrasound and X-ray imaging were available on-site. A non-medically managed welfare service was also provided and offered guests a warm, sheltered safe space. For guests requiring transfer off-site, ambulances were dedicated to the event and on standby to be dispatched.

### Inclusion Criteria

All guests, volunteers, staff, and performers who attended a medical facility from 8:00am Wednesday June 22, 2022 through 5:00pm Monday June 27, 2022 were included.

### Data Collection, Extraction, and Analysis

On arrival at one of the on-site medical centers, patients were registered on an EPR system, recording their key demographics. At discharge, the system was updated with a diagnosis, any treatment provided, and where the patient was discharged. This was compiled using 120 pre-determined diagnosis codes, 92 treatment codes, and 12 discharge locations which administrators at the festival recorded. If none of the pre-determined codes were suitable, the record could be coded as “non classifiable,” and if appropriate, a record could be assigned multiple unique codes.

After the festival, an anonymized version of the EPR system was obtained and imported into Microsoft Excel Version 16.72 (Microsoft Corporation; Redmond, Washington USA). This included all diagnosis, treatment, and discharge location data, however the only demographic data obtained were year of birth, gender, whether the patient was working at the festival, and the time and date of the presentation. Year of birth was used to calculate an approximate age, and these data were subsequently grouped into 10-year intervals for analysis.

Diagnoses were categorized by researchers using a modified version of Ranse and Hutton’s Minimum Data Set.^32^ Illness, injury, environment, mental health, not classifiable, and nothing abnormal detected were used as broad categories. A further 51 subcategories were reported to provide a detailed breakdown of the diagnoses. Researchers matched the 120 pre-determined coded diagnoses to the 51 subcategories. For example, the EPR category “assault – physical” was matched to the “assault” subcategory and “burns” was matched to the “burns” subcategory. Where there was uncertainty, consensus decisions were reached following discussions between the two authors. The anatomical location of any injury was not recorded within the EPR, and as a result, is not presented. Treatment provided at the festival and the location of discharge are presented as they were recorded within the EPR. No other identifiable information was obtained.

### Missing Data and Validity

Not classifiable was only recorded a small number of times (24/2,995; 0.80%) out of all the diagnosis codes and it was not recorded for treatment or discharge locations. This is presented within the results.

A small number of patients (40/2,828; 1.41%) had no recorded year of birth so they were excluded from the average age statistic; however, their record was included in all other outputs and results. No patient record had a missing presentation time, presentation date, gender, or worker status.

Any patient transferred internally between on-site medical facilities had two electronic medical records created, one at each site. To ensure none of the data were duplicated, only the second record was used, except for the initial time of presentation and any treatment that had been provided. The time of presentation on the first record was exclusively used and any treatment data were manually merged with the second record.

Some patients were attended by first responders on foot, bicycle, or in an ambulance. Due to the nature of music festivals, first responder teams recorded patient encounters on paper proformas. The time and date these proformas were uploaded to the electronic record system were recorded as the presentation time rather than when the responder first attended to the patient. As a result, any patients who were attended to by first responders were excluded from any date or time analysis, although the record was reported in all other parameters. This represented a very small sample of the population (121/2,828; 4.28%).

Ethical approval for this study was granted by the University of Leeds, School of Medicine Research Ethics Committee (Leeds, England; MREC 22-030).

## Results

### Demographics

Over the festival’s six days, 2,828 patients were attended to by on-site medical teams. There was an approximately even split between females (1,509; 53.36%) and males (1,303; 46.06%), with a smaller number of guests describing themselves as gender neutral (3; 0.11%) or another gender (13; 0.46%). The ages of those presenting ranged from 0 to 86 with a mean age of 34.20 (SD = 13.52) years. Almost one-half of the patients were aged between 21 and 30 (Table 1).

**Table 1.** Age Distribution of Patients

Age	Number	Percentage
0-10	71	2.51%
11-20	101	3.57%
21-30	1,264	44.70%
31-40	629	22.24%
41-50	320	11.32%
51-60	231	8.17%
61-70	140	4.95%
71-80	28	0.99%
81-90	4	0.14%
Not Recorded	40	1.41%

The patient presentation rate (PPR) was 13.47 per 1,000 attendees.

### Time and Date of Presentation

**Figure 1.**
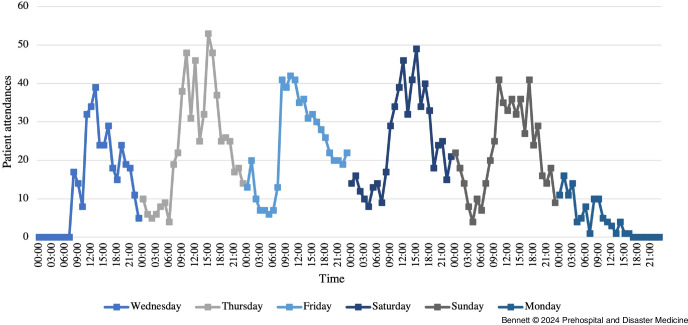
Number of Patient Attendances per Hour Throughout the Whole Festival in One-Hour Time Periods Commencing from the Displayed Value.

**Figure 2.**
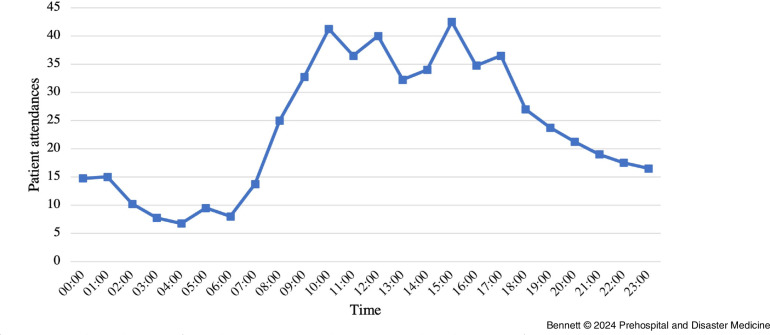
Average Number of Patient Attendances per Hour during Days when there were Amplified Performances in One-Hour Periods Commencing from the Displayed Value.

There were significantly more patient presentations during the show days with amplified music performances. On the two days without, there were 109 and 333 patient attendances; however, during the four days with amplified performances, there was very little variation with between 533 and 593 each day (Figure 1). In comparison, there was a large variation in the time-of-day patients presented. Over these four days, the most common period for patients to present was from 3:00pm through 3:59pm, with an average of 42.5 patients on each day. This was significantly higher than the least common time from 6:00am through 6:59am when there was only an average of eight patients per day (Figure 1). In the same period, at least one-half of the patients who presented each day presented from 9:00am through 5:59pm, despite the headline acts often performing to 11:30pm (Figure 2).

### Diagnosis

The 2,828 patient records recorded a total of 2,995 diagnoses, equating to an average of 1.06 diagnoses per record. Only a small number of patients (28; 0.99%) had more than two diagnoses and only one patient (0.04%) had more than five. The most diagnosed condition was joint injuries which was recorded 208 times and represented 6.94% of all diagnoses. This was followed by gastrointestinal conditions (201; 6.71%) and blisters (200; 6.68%); Table 2.

**Table 2.** Number of Diagnoses Recorded by Category and the Associated Percentage of the Total Number of Diagnoses

Category	Subcategory	Count	Percentage
Environment	Assault	4	0.13%
Environment	Burns	66	2.20%
Environment	Crush Injury	5	0.17%
Environment	Other	0	0.00%
Environment	Exposure & Hypothermia	17	0.57%
Environment	Dehydration	26	0.87%
Environment	Heat Exhaustion	29	0.97%
Environment	Sunburn	19	0.63%
Environment	Insect Bites and Stings	49	1.64%
Environment	Intoxication	164	5.48%
Illness	Allergy	64	2.14%
Illness	Cardiac Condition	27	0.90%
Illness	Dental Condition	81	2.70%
Illness	Dermatological Condition	94	3.14%
Illness	Endocrine Condition	16	0.53%
Illness	ENT Condition	119	3.97%
Illness	Gastrointestinal Condition	201	6.71%
Illness	Gynecological Condition	30	1.00%
Illness	Infective Illness	63	2.10%
Illness	Medication Issue	93	3.11%
Illness	Neurological Condition	105	3.51%
Illness	Obstetric Condition	9	0.30%
Illness	Ophthalmological Condition	43	1.44%
Illness	Other	21	0.70%
Illness	Pre-Existing Medical Problem	19	0.63%
Illness	Respiratory Condition	120	4.01%
Illness	Sexual Health Condition	2	0.07%
Illness	Soft Tissue Infection	131	4.37%
Illness	Urological Condition	117	3.91%
Injury	Back Injury	33	1.10%
Injury	Blister	200	6.68%
Injury	Foreign Body in Ear	10	0.33%
Injury	Foreign Body in Eye	33	1.10%
Injury	Fracture	43	1.44%
Injury	Head Injury	44	1.47%
Injury	Joint Dislocation	13	0.43%
Injury	Joint Injury	208	6.94%
Injury	Laceration	139	4.64%
Injury	Muscle/Tendon Injury	148	4.94%
Injury	Eye Injury	6	0.20%
Injury	Eye Irritation	120	4.01%
Injury	Chest Injury	13	0.43%
Injury	Soft Tissue Injury	149	4.97%
Mental Health	Acute Confusional State	3	0.10%
Mental Health	Acute Psychosis	3	0.10%
Mental Health	Anxiety	39	1.30%
Mental Health	Depression	3	0.10%
Mental Health	Suicidal Ideation	7	0.23%
Mental Health	Drug-Induced Psychiatric Condition	2	0.07%
Not Classifiable	Not Classifiable	24	0.80%
Nothing Abnormal Detected	Nothing Abnormal Detected	21	0.70%

Of the categorical data, almost one-half of all diagnoses were an illness (1,355; 45.24%). This was closely followed by injury (1,159; 38.70%) and environment (379; 12.65%). A smaller number of diagnoses were categorized as mental health (57; 1.90%) or nothing abnormal detected (21; 0.70%). Only 24 diagnoses were recorded as unclassifiable (0.80%). Coronavirus disease 2019 (COVID-19) was diagnosed in only a minority of patients (11; 0.37%).

**Figure 3.**
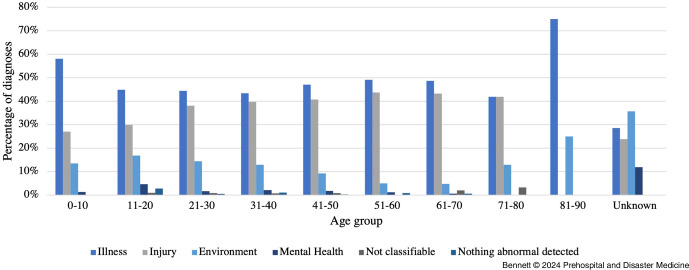
The Relative Percentage Proportion of Each Diagnosis Category by Age Group.

There was a large variation in the relative proportion of diagnoses of each category for different age groups (Figure 3). Young children were diagnosed with more illness and had less injury compared to the overall study population. Excluding the 81-91 age group, which only had four patients, illness as a proportion of diagnosis categories was most common in the 0-10 years group (43; 58.11%). Meanwhile, injury accounted for a smaller proportion (20; 27.03%) of diagnoses in young children aged 0-10 years despite accounting for over 40% of all diagnoses in age groups 41-50, 51-60, 61-70, and 71-80 years (Figure 3). Mental health diagnoses were highest in the unknown age group (5; 11.93%)

Just under one-third of the presentations to medical services were from staff working at the festival (772; 27.30%). The most diagnosed condition in this group was soft tissue injuries, which was recorded 58 times and represented 7.20% of all diagnoses from this group. This was followed by joint injuries (55; 6.83%) and blisters (51; 6.33%).

### Treatment

A total of 3,711 treatment interventions were recorded for the 2,828 patients, equating to an average of 1.31 interventions per patient. The most common intervention was verbal advice and guidance, which was recorded as being given to 1,090 patients (38.54%). The second most common intervention was prescribing a medication to take away (552; 19.52%), followed by wound dressing (268; 9.48%) and administering oral medication (241; 8.52%).

Of the anesthetic interventions, the most common was local anesthetic infiltration (7; 0.25%), insertion of an airway adjunct (4; 0.14%), and general anesthetic (1; 0.04%). No patients required cardiopulmonary resuscitation and there were no recorded deaths during the festival.

### Discharge

Most patients (2,563; 90.66%) were discharged immediately back to the festival from medical care. A minority of patients (64; 2.26%) were transferred to an off-site hospital, 62 (2.19%) by road ambulance, and two (0.07%) by air ambulance. Of the patients who were transferred to an off-site hospital, the mean age was higher than that of the whole study population (41.09; SD = 16.31). There was approximately an even split of males (33; 51.56%) and females (31; 48.44%). The transport-to-hospital rate (TTHR) was 0.30 patients per 1,000 festival attendees.

The 64 patients who were transferred had a total of 82 diagnoses. The most common diagnosis in patients transferred to a hospital was gastrointestinal conditions, which was recorded 10 times and represented 12.20% of all diagnoses from this group. This was followed by neurological conditions (10; 12.20%) and fractures (9; 10.98%). Almost three-quarters of the diagnoses were categorized as an illness (56; 68.29%). The remaining diagnoses were categorized between injury (19; 23.17%), environment (5; 6.10%), mental health (1; 1.22%), and non-classifiable (1; 1.22%). Only five patients (6.10%) who were transferred to an off-site hospital had been diagnosed as being intoxicated.

Of the remaining patients, three (0.11%) made their own transport to a hospital after having been referred and three further patients (0.11%) declined a referral to a hospital and subsequently self-discharged. A small number of patients (18; 0.67%) reported that they would attend a hospital after leaving the festival site without a referral.

A small number were discharged to a welfare organization (48; 1.70%), the police (5; 0.18%), or an alternative organization (4; 0.15%), which included the event management team and a charity for victims of sexual assault. Twenty-seven patients (0.95%) were discharged home and left the festival. A minority of patients self-discharged (92; 3.25%), including the three (0.11%) who refused a hospital referral and four (0.15%) were unknown.

### Intoxicated Patients

A small proportion of patients (164; 5.48%) were diagnosed as being intoxicated with either drugs, alcohol, or both drugs and alcohol. The average age of these patients was slightly lower than the overall study average (30.36; SD = 9.12) and contained a higher proportion of male patients (102; 62.20%). Twenty-three patients had an additional diagnosis and two patients had two additional diagnoses. These additional diagnoses varied significantly, however the most common of which were cardiac conditions (3; 11.11%) and neurological conditions (3; 11.11%). The discharge locations of intoxicated patients varied notably in comparison to the overall study. Only 105 patients (64.02%) were discharged back to the festival site, significantly lower than the study population of 90.66%. Furthermore, a higher percentage of these patients (17; 10.37%) self-discharged compared to the study population of 3.25%. Although some patients were transferred to an off-site hospital (5; 3.05%), many patients were discharged to the welfare tent (34; 20.73%). This provided a safe alternative for their recovery. The remaining intoxicated patients were discharged to the police (2; 1.22%) or were unknown (1; 0.61%).

### Patients Aged 65 and Over

Eighty-eight patients (3.11%) who sought medical attention were aged 65 and over. They were diagnosed with 93 conditions, of which the most common were blisters (9; 9.68%), soft tissue infections (8; 8.60%), dermatological conditions (7; 7.53%), and neurological conditions (7; 7.53%). Neurological and dermatological conditions were the joint third most common diagnoses in this sub-population, however, in the total festival population, they were the thirteen and fourteenth most common diagnoses, respectively.

Whilst the majority of patients aged 65 and over returned to the festival site (72; 81.82%), it was notably less than the overall festival population. Furthermore, a much higher proportion of patients were transported to hospital (5; 5.68%).

## Discussion

Whilst mass-gathering events are frequently attended by a wide variety of age groups, music festivals are often targeted towards young adults.^33^ Despite this, Glastonbury Festival attracts a wider age range than most music festivals.^24,25^ These results showed that patients aged 65 years and over were diagnosed with a different frequency of conditions in comparison to the study population and a higher percentage of these patients required transporting to an off-site hospital.

Similarly, these results showed young children were also diagnosed with a different frequency of conditions compared to the study population. Children less than 10 years of age, specifically, had a much higher rate of diagnoses within the illness category and less injury compared to the study population. Whilst previous literature has reviewed the care of children at music festivals, very little is published on the under 10 years age group.^3,34^ Despite this paper providing insight into the varying care required by these two subpopulations, the small numbers of these patients make it challenging to draw any firm conclusions. Similar music festivals which are attended by young children and guests over the age of 65 should now be evaluated to determine if similar results are uncovered. If these results are replicated, event planners may be able to better prepare services to cater for these potentially forgotten population groups.

Whilst the festival has been running for almost 50 years, it has never been impacted by a global pandemic.^35^ Mass-gathering events have been previously associated with outbreaks of infectious diseases, including an outbreak of E. Coli O157 at a previous Glastonbury Festival.^36–38^ The 2022 festival was the first festival held at the site since the start of the COVID-19 pandemic. As guests were not required to be vaccinated or carry out testing prior to the event, it was uncertain the implications COVID-19 may have on medical presentations.^39^ With only 11 patients (0.37%) being diagnosed with COVID-19 and none of these patients required conveying to an off-site hospital, these data suggest COVID-19 had a minimal impact on the festival. Given that not all attendees were tested, the overall incidence was likely higher, however it is reassuring that they did not need to seek medical attention. In keeping with other published literature, it would suggest music festivals can continue to be carried out safely, despite the risks of COVID-19.^40,41^

The TTHR of mass-gathering events varies greatly across different types of events as well as between events within similar categories.^42^ Due to the unique performing arts event that Glastonbury is, it is hard to compare to similar events; however, the TTHR of 0.30 per 1,000 attendees is in line with other music festivals. For example, a rate of 0.38 was recorded at Europe’s largest electronic dance music festival in Boom, Belgium in 2019.^43^ Several factors are likely responsible for this, including the demographics of the population, the relatively stable climate conditions in the United Kingdom, and the range of medical facilities on-site, including imaging. Previous research has demonstrated the benefit of on-site radiological services in reducing the burden of festivals on local health services.^14,19^ Given ultrasound and X-ray interventions were performed 178 times at the festival, it is likely on-site imaging also reduced the TTHR at the 2022 Glastonbury Festival.

The presence of on-site medical services has been suggested to impact attendees’ perception of recreational drug use risk.^44^ Glastonbury Festival has an extensive medical provision, and this may paradoxically increase rates of consumption of recreational drugs and alcohol as attendees perceive the risk to be less. Whilst this may be the case, this study’s data suggest intoxication with drugs and alcohol only minimally contributed to the medical burden of the festival. Only 164 patients (5.48%) were diagnosed as being intoxicated with alcohol, drugs, or both alcohol and drugs and only five of these patients (3.05%) required transfer to an off-site hospital. Despite this, the impact of alcohol and recreational drugs may not be fully explained by these data. Whilst patients may not have been diagnosed as being intoxicated at the time of presentation, consumption of alcohol or recreational drugs may have contributed to a later presentation. The negative short-term health implications of recreational drugs and alcohol are well-documented, and given the festival environment, this may be further exaggerated.^45^

It was also noted the positive impact the welfare services had at Glastonbury Festival 2022 with over one-fifth of patients (34; 20.73%) who had been diagnosed as being intoxicated being discharged there. This in turn likely reduced the rate of off-site transfers and should be considered by other mass-gathering events as a safe alternative to discharge these patients.

## Limitations

The authors had to work with the data made available by the charity providing the medical services on-site. The steps taken to extract data, assign diagnostic labels, and deal with the missing and unclassifiable data from the records are described above. However, the authors had no way of knowing how complete or accurate the EPR data were. The charity reports that it takes great care to ensure records are opened and accurately compiled for all those who present, despite the difficult working circumstances described in the Setting section above. Regular checks were made by charity staff on the completeness of records in real time, and as reported above, levels of missing data in the records were low.

This study only describes one event retrospectively. Given the nature of music festivals and the many known factors that can affect patient presentations, there is likely significant variation between different festivals and different years at the same festival. To better understand the medical epidemiology of Glastonbury Festival, it would be more effective to undertake a longitudinal review of the festival.

Another limitation of this study was that by using anonymized patient data, authors were unable to review which patients reattended on-site medical services. As a result, it is possible within the data set that some patients may have visited medical centers multiple times for the same issue. In future research, it would provide an interesting insight to determine what type of patients these are, and subsequently, if on-site medical services can be optimized to reduce the chance of patients reattending.

Another issue of using anonymized patient data was that it prevented the tracing of patients after they had been discharged. It would have been particularly insightful to obtain further details of patients who had been discharged to secondary care to determine their definitive diagnosis.

## Conclusion

This study highlights the varied medical presentations, diagnoses made, and necessary treatment at the 2022 Glastonbury Festival. Minor conditions such as joint injuries, gastrointestinal conditions, and blisters are responsible for many presentations. Contrary to popular belief, only a small number of patients presented to medical staff or required medical treatment as a result of being intoxicated. Most patients only required mild or non-invasive interventions and could safely be discharged back to the festival.
